# The incidence, risk factors and predictive nomograms for early death of lung cancer with synchronous brain metastasis: a retrospective study in the SEER database

**DOI:** 10.1186/s12885-021-08490-4

**Published:** 2021-07-16

**Authors:** Heng Shen, Gang Deng, Qianxue Chen, Jin Qian

**Affiliations:** 1 Department of neurosurgery, Suizhou Hospital, Hubei University of Medicine, 60 Longmen Street, Suizhou, 441399 Hubei China; 2 Department of Neurosurgery, Renmin Hospital of Wuhan University, 238 Jiefang Road, Wuhan, 430060 Hubei China

**Keywords:** Lung cancer, Brain metastases, Nomogram, Early death

## Abstract

**Background:**

The prognosis of lung cancer with synchronous brain metastasis (LCBM) is very poor, and patients often die within a short time. However, little is known about the early mortality and related factors in patients with LCBM.

**Methods:**

Patients diagnosed with LCBM between 2010 and 2016 were enrolled from the Surveillance, Epidemiology, and End Result (SEER) database. Univariate and multivariate logistic regression analysis were used to identify significant independent prognostic factors, which were used to construct nomograms of overall and cancer-specific early death. Then, the prediction ability of the model was verified by receiver operating characteristic (ROC) curve. At last, the clinical application value of the model was tested through decision curve analysis (DCA).

**Results:**

A total of 29,902 patients with LCBM were enrolled in this study. Among them, 13,275 (44.4%) patients had early death, and 11,425 (38.2%) cases died of lung cancer. The significant independent risk factors for overall and cancer-specific early death included age, race, gender, Gleason grade, histological type, T stage, N stage, bone metastasis, liver metastasis and marital status, which were used to construct the nomogram. The ROC curve demonstrated good predictive ability and clinical application value. The areas under the curve (AUC) of the training group was 0.793 (95% CI: 0.788–0.799) and 0.794 (95% CI: 0.788–0.799), in the model of overall and cancer-specific early death respectively. And the AUC of the validation group were 0.803 (95% CI: 0.788–0.818) and 0.806 (95% CI: 0.791–0.821), respectively. The calibration plots of the model showed that the predicted early death is consistent with the actual value. The DCA analysis indicated a good clinical application value of this model.

**Conclusions:**

We established a comprehensive nomogram to predict early death in lung cancer patients with synchronous brain metastases. Nomograms may help oncologists develop better treatment strategies, such as clinical trials and hospice care.

**Supplementary Information:**

The online version contains supplementary material available at 10.1186/s12885-021-08490-4.

## Introduction

Brain metastases (BM) are the most common malignant tumor in the central nervous system [1, 2]. It is reported that the incidence of brain metastases is 10 times higher than that of primary malignant brain tumors [3]. Most brain metastases progress rapidly, with an average survival time of 13 months [4]. Lung cancer is the leading cause of brain metastasis, accounting for more than 80% [5].

Currently, there is no reliable treatment for lung cancer with synchronous brain metastasis (LCBM). Surgical treatment is not recommended for patients with LCBM because it has no significant impact on the long-term prognosis, although the symptoms are temporarily relieved [6]. In comparison, intracranial tumor biopsy is the gold standard for the diagnosis of LCBM, which can not only determine the nature of intracranial lesions, but determine their source. The combination of radiotherapy and targeted therapy has gradually become the current treatment of primary lung cancer [7]. And regular MRI review was used to monitor the therapeutic effect. With the development of molecular biology, more and more abnormal signal transduction and tumor-driving genes have been found, and increasing targeted drugs have been designed to prolong the overall survival of patients [8–10]. However, it is difficult obtain effective drug concentration in cerebrospinal fluid, due to the existence of the blood-brain barrier (BBB) [11]. As a result, the treatment of lung cancer will be very poor once brain metastases occur.

Death within a short period of time after diagnosis is defined as early death. And many patients with LCBM die early death due to intracranial hypertension and tumor-related epilepsy [12]. An in-depth understanding of the relationship between tumor-related factors and early death may help us reveal the causes of early death in high-risk patients, and provide basis for further active treatment, clinical trial consideration and supportive treatment. However, few studies have focused on the early death of patients with LCBM. Little is known about the early mortality and related factors in patients with LCBM currently. Thus, it’s of great significance to identify the risk factors of early death for prognostic evaluation and clinical treatment guidance in patients with LCBM.

In this study, patients with LCBM in Surveillance, Epidemiology, and End Results (SEER) database were included as the research objects to evaluate the incidence of early death and explore the risk factors of early death (≤3 months). In addition, we developed a module containing prognostic factors to predict the early mortality of patients with LCBM.

## Methods

### Patients

The SEER project of the National Cancer Institute provides data on cancer incidence and survival rate of 28% of the population in the United States (http://seer.cancer.gov) [13]. In this study, data of malignant lung and bronchial cancer patients with synchronous brain metastasis was extracted from the SEER database (2010–2016) by the SEER*Stat software version 8.3.8 (reference number: 17293-November 2019). The study cohort included the following histological codes from the third edition of the International Classification of Diseases for Oncology (ICD-O-3): large cell carcinoma (LCLC): 8012, 8014; squamous cell carcinoma (SQLC):8070, 8071, 8072, 8073, 8074, 8083; adenoma (AD): 8140, 8200, 8230, 8250, 8255, 8260, 8290, 8310, 8323; small cell carcinoma (SCLC): 8041, 8043, 8044, 8045 and ICD-O-3 site code C34.0–34.8. The exclusion criteria are as follows: (1) Patients without histological examination; (2) Patients without complete follow-up; (3) Patients with missing or incomplete information about survival time, survival status, cause of death, or other important characteristics; (4) Patients not primary. In addition to topography and morphology, it also includes clinical information such as gender, race, age, TN stage, insurance status, and marital status. The patients diagnosed in 2010–2015 were used as a training cohort to develop a nomogram, and the patients diagnosed in 2016 were selected as the internal validation group.

28 patients with LCBM from Renmin Hospital of Wuhan University were included in the study. The definition of cancer-specific early death in the hospital cohort was: death within 3 months after initial diagnosis of primary lung cancer with brain metastases. This retrospective study of the hospital cohort was approved by the ethics committee of Renmin Hospital of Wuhan University in accordance with the ethical standards approved by the Helsinki declaration.

### Statistical analysis

Categorized data were described by numbers and percentages (N, %). Early death, defined as death within 3 months after diagnosis, was the endpoint of interest for this study. Histogram and pie chart were drawn with SPSS25 (IBM Inc., Chicago, IL, USA). Univariate and multivariate logistic regression models were performed using SPSS25 to determine variables that were significantly related to early death of patients with LCBM. Two-tailed *P* values less than 0.05 were considered statistically significant. All statistical analysis below was performed using the R programming language and environment (http://www.r-project.org/). The “regplot” software package was used to construct a nomogram of independent factors predicting early death of patients with LCBM [14]. For calibration, the nomogram predicted probabilities were contrasted with the actual probabilities by bootstrapping with 1000 resamples. The receiver operating characteristic (ROC) curve was used to judge discrimination. The higher the area under the curve (AUC) was, the better the accuracy would be. AUC values vary from 0.5 to 1.0, where 0.5 represents random chance, and 1.0 represents full compliance. And AUC value greater than 0.7 means a reasonable estimate [15]. Decision curves analysis (DCA) was used to assess the clinical benefit and utility of the model. DCA is one way to evaluate the clinical benefit of alternative models, and is applied to nomograms by quantifying the net benefit under different threshold probabilities. The curves of the treatment plan (representing the highest clinical cost) and no treatment plan (representing no clinical benefit) for all patients are drawn as two references [16, 17].

## Result

### Demographic and clinical characteristics of lung cancer patients with synchronous brain metastasis

**Fig. 1:**
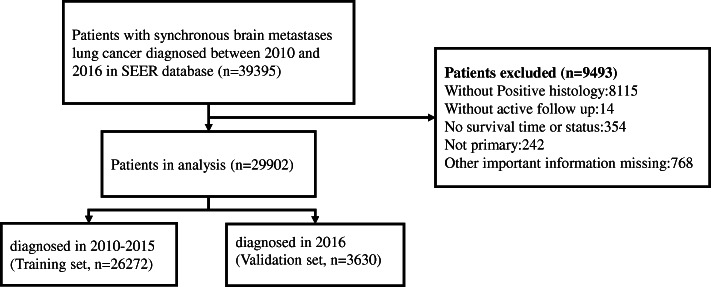
The flowchart of patient selection from SEER database

This study included 29,902 patients diagnosed with synchronous brain metastases of lung cancer from 2010 to 2016 in the SEER database (Fig. 1). Table 1 listed the demographic and clinicopathological characteristics of patients in the training cohort (*n* = 26,272) and validation cohort (*n* = 3630). In general, most of the patients were over 40 years old, and the male was slightly more than the female. 79.5% of the cases were white and 12.2% were black. Most of them were adenocarcinoma (51.6%), and SQCC, SCLC and LCLC accounted for 10.6, 17.0 and 2.3%, respectively. Gleason grade III lung cancer was significantly higher than other grades. Some cases were accompanied with liver metastases (20.7%) or bone metastases (33.5%). Very few patients received surgical treatment (3.2%), a small number of patients received radiotherapy (21.1%), and about half of the patients received chemotherapy (56.1%). There was no significant difference in composition between the training group and the validation group.

**Table 1:** Demographic and tumor characteristics of lung cancer patients with brain metastases

Variable	All subjects	[cases (%)]	Training cohort	[cases (%)]	Validation cohort	[cases (%)]
Total	29,902		26,272		3630	
**Gender**						
Male	15,596	52.2	13,741	52.3	1855	51.1
Female	14,306	47.8	12,531	47.7	1775	48.9
**Age**						
< 40	252	0.8	212	0.8	40	1.1
40–49	1648	5.5	1479	5.6	169	4.7
50–59	7149	23.9	6308	24.0	841	23.2
60–69	10,447	34.9	9150	34.8	1297	35.7
70–79	7751	25.9	6775	25.8	976	26.9
> =80	2655	8.9	2348	8.9	307	8.5
**Race**						
White	23,759	79.5	20,947	79.7	2812	77.5
Black	3643	12.2	3190	12.1	453	12.5
Other	2500	8.4	2135	8.1	365	10.1
**Histology**						
AD	15,427	51.6	13,467	51.3	1960	54.0
SQCC	3164	10.6	2770	10.5	394	10.9
LCLC	675	2.3	601	2.3	74	2.0
SCLC	5087	17.0	4480	17.1	607	16.7
Other	5547	18.6	4952	18.8	595	16.4
**Gleason grade**						
I	463	1.5	409	1.6	54	1.5
II	2677	9.0	2398	9.1	279	7.7
III	7821	26.2	6927	26.4	894	24.6
IV	1046	3.5	954	3.6	92	2.5
Unknown	17,895	59.8	15,584	59.3	2311	63.7
**T Stage**						
T0	292	1.0	266	1.0	26	0.7
T1	3244	10.8	2803	10.7	441	12.1
T2	7386	24.7	6441	24.5	945	26.0
T3	6534	21.9	5826	22.2	708	19.5
T4	8802	29.4	7674	29.2	1128	31.1
Tx	3644	12.2	3262	12.4	382	10.5
**N Stage**						
N0	6446	21.6	5675	21.6	771	21.2
N1	2531	8.5	2213	8.4	318	8.8
N2	13,441	45.0	11,892	45.4	1549	42.7
N3	5773	19.3	4994	19.0	779	21.5
Nx	1711	5.7	1498	5.7	213	5.9
**Bone Met**						
Yes	10,024	33.5	8750	33.3	1274	35.1
None	19,306	64.6	17,006	64.7	2300	63.4
Unknown	572	1.9	516	2.0	56	1.5
**Liver Met**						
Yes	6189	20.7	5390	20.5	799	22.0
None	22,971	76.8	20,226	77.0	2745	75.6
Unknown	742	2.5	656	2.5	86	2.4
**Surgery**						
Yes	961	3.2	858	3.3	103	2.8
None/ Unknown	28,941	96.8	25,414	96.7	3527	97.2
**Chemotherapy**						
Yes	16,767	56.1	14,762	56.2	2005	55.2
None/ Unknown	13,135	43.9	11,510	43.8	1625	44.8
**Radiotherapy**						
Yes	6307	21.1	5497	20.9	810	22.3
None/ Unknown	23,595	78.9	20,775	79.1	2820	77.7
**Insurance**						
Yes	28,191	94.3	24,723	94.1	3468	95.5
None	1221	4.1	1128	4.3	93	2.6
Unknown	490	1.6	421	1.6	69	1.9
**Marital**						
Single	13,326	44.6	11,643	44.3	1683	46.4
Married	15,424	51.6	13,608	51.8	1816	50.0
Unknown	1152	3.9	1021	3.9	131	3.6

### Mortality of early death

**Fig. 2:**
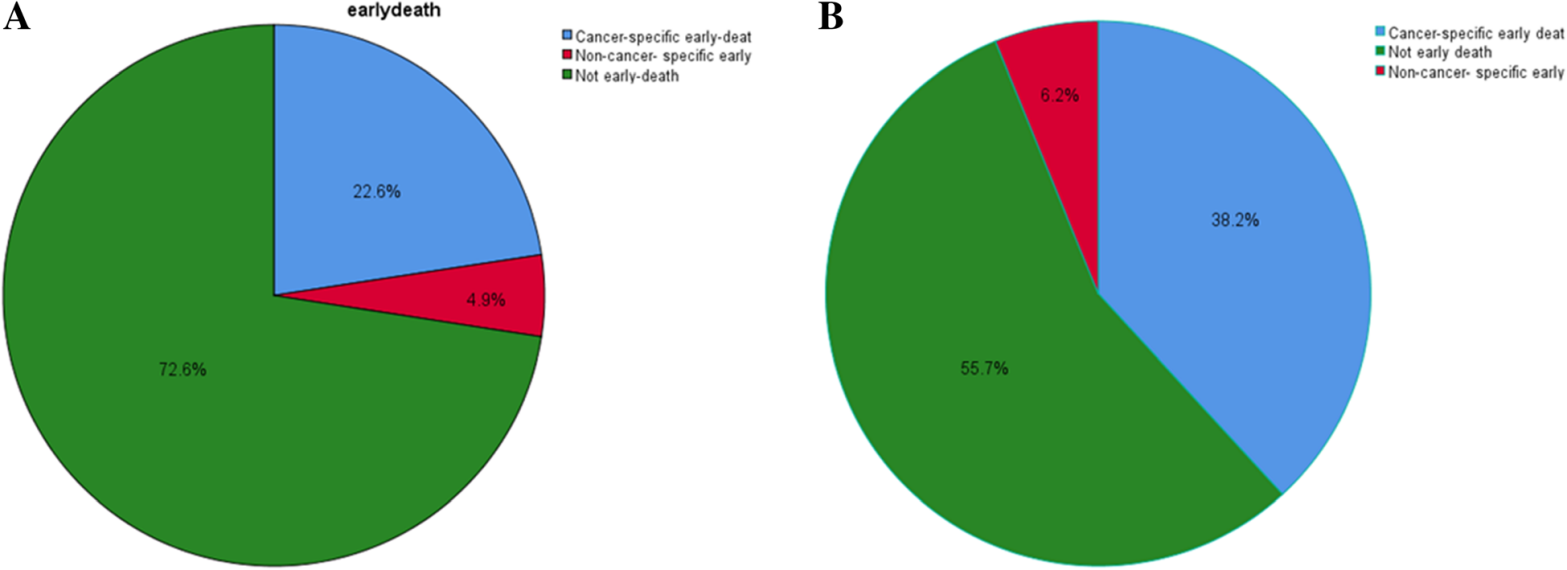
Distribution of the incidence of overall and cancer-specific early death in all lung cancer patients (**A**) and lung cancer with synchronous brain metastasis patients (**B**)

**Fig. 3:**
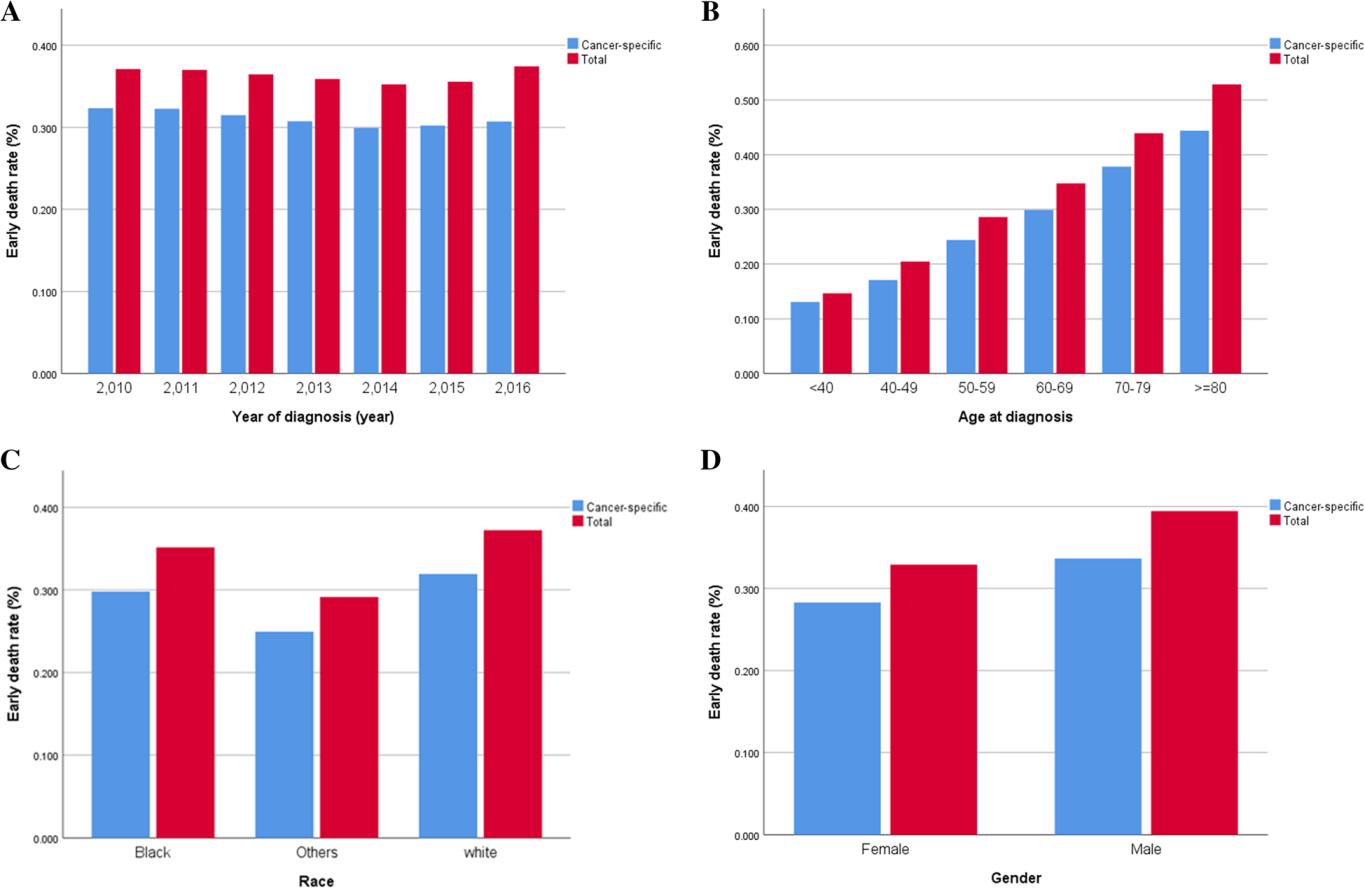
Rate of overall and cancer-specific early death in lung cancer with synchronous brain metastasis patients stratified by year of diagnosis (**A**), age at diagnosis (**B**), race (**C**) and gender (**D**)

Among all lung cancer patients, 27.5% had early death, and 22.6% of them were caused by lung cancer (Fig. 2A). However, the early mortality of patients with LCBM was 44.4% (13275), and 38.2% (11425) of them were caused by lung cancer (Fig. 2B). From 2010 to 2016, the early mortality of patients with LCBM remained stable (Fig. 3A). The early mortality increased significantly with age, was slightly higher in white people than in other ethnic groups, and higher in male than in female (Fig. 3B, C, D).

### Identifying independent factors for early death

Univariate and multivariate logistic regression were used to analyze the risk factors of early death in patients with LCBM in the SEER training cohort. The results of univariate and multivariate analysis were shown in Table 2 and Table 3. In univariate analysis, most clinical and pathological characteristics such as gender, race, age at diagnosis, Gleason grade, histology, T stage, N stage, bone metastasis, liver metastasis and marital status were related to the probability of overall early death. All significant factors were included in the multivariate analysis. And multivariate analysis showed that gender, race, age at diagnosis, Gleason grade, histology, T stage, N stage, bone metastasis, liver metastasis and marital status were independent risk factors for predicting overall early death in patients with LCBM. The result of cancer-specific early death is consistent with that of overall early death.

**Table 2:** Univariate logistic regression analysis of the training dataset

Variable	Overall early-death			Cancer-specific early-death		
	OR	95% CI	P	OR	95% CI	P
**Gender**						
Male	Ref			Ref		
Female	0.745	0.709–0.784	< 0.001	0.773	0.734–0.815	< 0.001
**Age**						
< 40	Ref			Ref		
40–49	1.57	1.046–2.356	0.03	1.441	0.942–2.205	0.092
50–59	2.43	1.645–3.589	< 0.001	2.215	1.473–3.33	< 0.001
60–69	3.205	2.173–4.727	< 0.001	2.921	1.946–4.385	< 0.001
70–79	4.687	3.176–6.917	< 0.001	4.169	2.776	< 0.001
> =80	6.824	4.599–10.126	< 0.001	5.571	3.69–8.411	< 0.001
**Race**						
White	Ref			Ref		
Black	0.907	0.839–0.981	0.014	0.891	0.821–0.966	0.005
Other	0.695	0.630–0.766	< 0.001	0.705	0.637–0.781	< 0.001
**Histology**						
AD	Ref					
SQCC	1.784	1.642–1.939	< 0.001	1.7	1.561–1.85	< 0.001
LCLC	1.238	1.045–1.467	0.014	1.228	1.031–1.463	0.022
SCLC	1.192	1.111–1.28	< 0.001	1.156	1.074–1.245	< 0.001
Other	1.568	1.466–1.676	< 0.001	1.433	1.337–1.535	< 0.001
**Gleason grade**						
I	Ref			Ref		
II	1.121	0.885–1.421	0.344	1.17	0.912–1.502	0.217
III	1.814	1.448–2.272	< 0.001	1.813	1.43–2.298	< 0.001
IV	1.682	1.302–2.172	< 0.001	1.731	1.324–2.264	< 0.001
Unknown	1.568	1.256–1.958	< 0.001	1.519	1.202–1.921	< 0.001
**T Stage**						
T0	Ref			Ref		
T1	0.96	0.722–1.276	0.779	1.352	0.969–1.886	0.076
T2	1.315	0.997–1.734	0.053	1.928	1.393–2.669	< 0.001
T3	1.674	1.269–2.207	< 0.001	2.384	1.722–3.3	< 0.001
T4	1.826	1.387–2.406	< 0.001	2.648	1.915–3.661	< 0.001
Tx	1.925	1.454–2.548	< 0.001	2.561	1.844–3.557	< 0.001
**N Stage**						
N0	Ref			Ref		
N1	0.93	0.838–1.034	0.18	0.961	0.86–1.072	0.474
N2	1.203	1.126–1.286	< 0.001	1.226	1.144–1.315	< 0.001
N3	1.154	1.065–1.25	< 0.001	1.2	1.105–1.304	< 0.001
Nx	1.553	1.383–1.745	< 0.001	1.454	1.289–1.64	< 0.001
**Bone Met**						
Yes	Ref			Ref		
None	0.728	0.690–0.768	< 0.001	0.728	0.689–0.769	< 0.001
Unknown	0.998	0.833–1.196	0.984	0.903	0.748–1.09	0.286
**Liver Met**						
Yes	Ref			Ref		
None	0.519	0.489–0.552	< 0.001	0.543	0.51–0.577	< 0.001
Unknown	0.771	0.654–0.909	0.002	0.796	0.673–0.942	0.008
**Insurance**						
Yes	Ref			Ref		
None	0.894	0.791–1.01	0.073	0.947	0.834–1.076	0.407
Unknown	1.156	0.920–1.451	0.213	1.119	0.883–1.417	0.352
**Marital**						
Single	Ref			Ref		
Married	0.805	0.764–0.847	< 0.001	0.824	0.781–0.869	< 0.001
Unknown	0.906	0.794–1.035	0.147	0.841	0.731–0.967	0.015

**Table 3:** Multivariate logistic regression analysis of the training dataset

Variable	Overall early-death			Cancer-specific early-death		
	OR	95% CI	P	OR	95% CI	P
**Gender**						
Male	Ref			Ref		
Female	0.742	0.703–0.783	< 0.001	0.773	0.731–0.817	< 0.001
**Age**						
< 40	Ref			Ref		
40–49	1.715	1.133–2.596	0.011	1.558	1.011–2.4	0.044
50–59	2.634	1.769–3.923	< 0.001	2.377	1.57–3.597	< 0.001
60–69	3.494	2.35–5.197	< 0.001	3.144	2.08–4.51	< 0.001
70–79	5.296	3.559–7.881	< 0.001	4.634	3.065–7.007	< 0.001
> =80	7.726	5.162–11.563	< 0.001	6.176	4.062–9.389	< 0.001
**Race**						
White	Ref			Ref		
Black	0.941	0.867–1.022	0.151	0.923	0.847–1.005	0.064
Other	0.728	0.657–0.806	< 0.001	0.733	0.659–0.816	< 0.001
**Histology**						
AD	Ref			Ref		
SQCC	1.555	1.425–1.697	< 0.001	1.477	1.351–1.614	< 0.001
LCLC	1.188	0.994–1.42	0.058	1.178	0.981–1.415	0.08
SCLC	0.975	0.901–1.056	0.541	0.96	0.885–1.043	0.336
Other	1.434	1.336–1.539	< 0.001	1.315	1.223–1.414	< 0.001
**Gleason grade**						
I	Ref			Ref		
II	1.106	0.865–1.413	0.423	1.146	0.887–1.482	0.297
III	1.669	1.322–2.108	< 0.001	1.673	1.31–2.136	< 0.001
IV	1.555	1.187–2.038	< 0.001	1.614	1.218–2.137	0.001
Unknown	1.446	1.148–1.800	0.002	1.412	1.109–1.799	0.005
**T Stage**						
T0	Ref			Ref		
T1	0.951	0.71–1.273	0.734	1.334	0.951–1.872	0.095
T2	1.221	0.919–1.624	0.169	1.787	1.283–2.489	0.001
T3	1.512	1.137–2.01	0.004	2.138	1.535–2.978	< 0.001
T4	1.662	1.252–2.208	< 0.001	2.389	1.717–3.323	< 0.001
Tx	1.675	1.255–2.236	< 0.001	2.247	1.608–3.141	< 0.001
**N Stage**						
N0	Ref			Ref		
N1	0.877	0.786–0.979	0.019	0.904	0.806–1.013	0.082
N2	1.13	1.053–1.212	0.001	1.146	1.065–1.232	< 0.001
N3	1.108	1.018–1.207	0.018	1.145	1.048–1.25	0.003
Nx	1.257	1.107–1.426	< 0.001	1.212	1.065–2.38	0.004
**Bone Met**						
Yes	Ref			Ref		
None	0.791	0.746–0.839	< 0.001	0.795	0.748–0.844	< 0.001
Unknown	0.82	0.665–1.011	0.063	0.742	0.597–0.921	0.007
**Liver Met**						
Yes	Ref			Ref		
None	0.566	0.529–0.605	< 0.001	0.594	0.555–0.635	< 0.001
Unknown	0.734	0.607–0.887	0.001	0.816	0.672–0.989	0.039
**Marital**						
Single	Ref			Ref		
Married	0.757	0.717–0.8	< 0.001	0.779	0.736–0.824	< 0.001
Unknown	0.897	0.781–1.03	0.122	0.836	0.723–0.965	0.015

### Nomogram construction

**Fig. 4:**
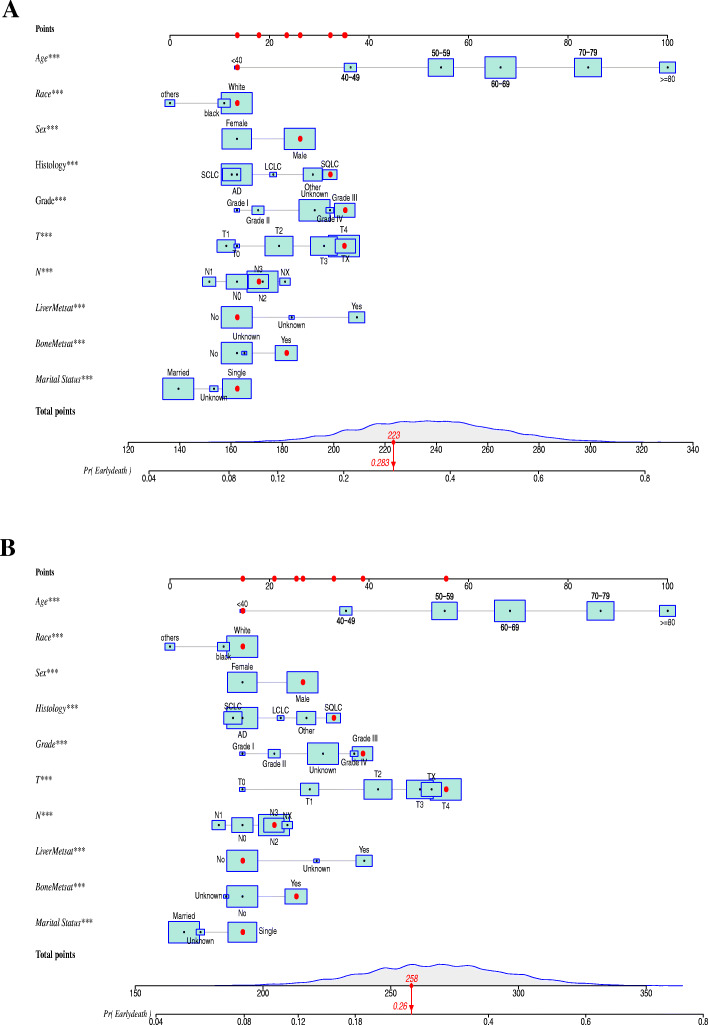
The predictive nomogram for the overall (**A**) and cancer-specific (**B**) early death of lung cancer with synchronous brain metastasis patients in the SEER database diagnosis between 2010 and 2015

Depending on the multivariate logistic regression analysis model, the risk factor prediction nomogram of the SEER cohort was determined. An example of using nomogram to predict the survival probability of a given patient was shown in Fig. 4A. The total number of points can be attached to the overall probability of early death by calculating each variable point. And most patients had a total score of between 200 and 350 in this study. The predicting nomogram of the probability of cancer-specific early death was shown in Fig. 4B.

### Nomogram validation

**Fig. 5:**
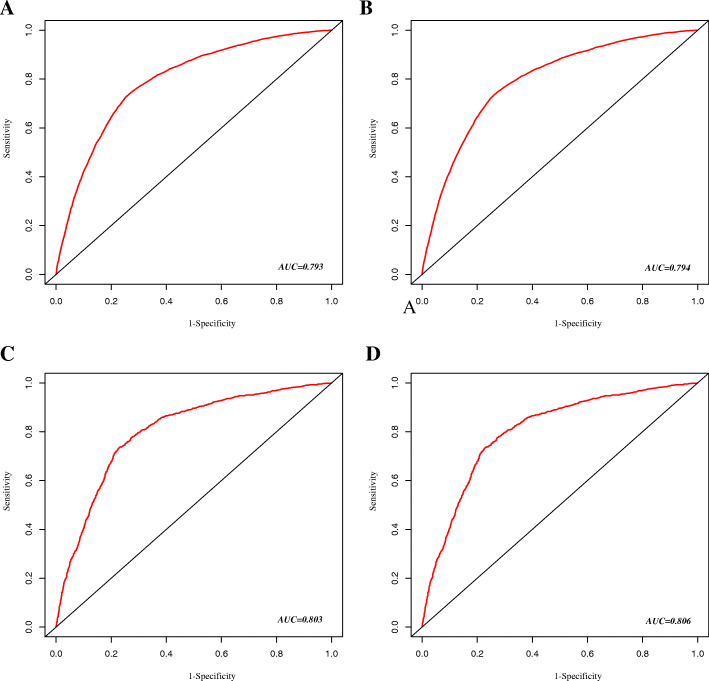
ROC curves for the nomogram. (**A**) The ROC curve for the overall early death nomogram in the SEER database diagnosis between 2010 and 2015; (**B**) The ROC curve for the overall early death nomogram in the SEER database diagnosis at 2016; (**C**) The ROC curve for the cancer-specific early death nomogram in the SEER database diagnosis between 2010 and 2015; (**D**) The ROC curve for the cancer-specific early death nomogram in the SEER database diagnosis at 2016

**Fig. 6:**
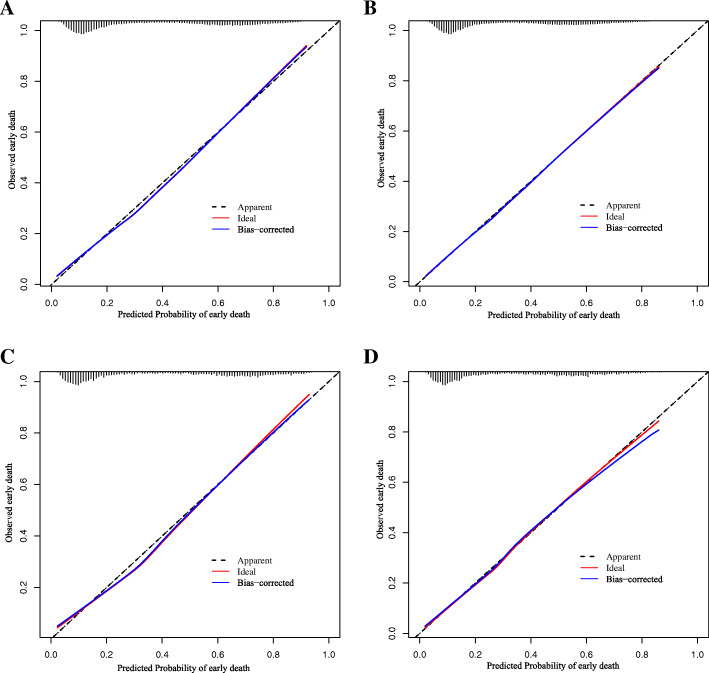
Calibration plots for the nomogram of (**A**) overall early death in the SEER database diagnosis between 2010 and 2015; (**B**) overall early death in the SEER database diagnosis at 2016; (**C**) cancer-specific early death in the SEER database diagnosis between 2010 and 2015; (**D**) cancer-specific early death in the SEER database diagnosis at 2016

**Fig. 7:**
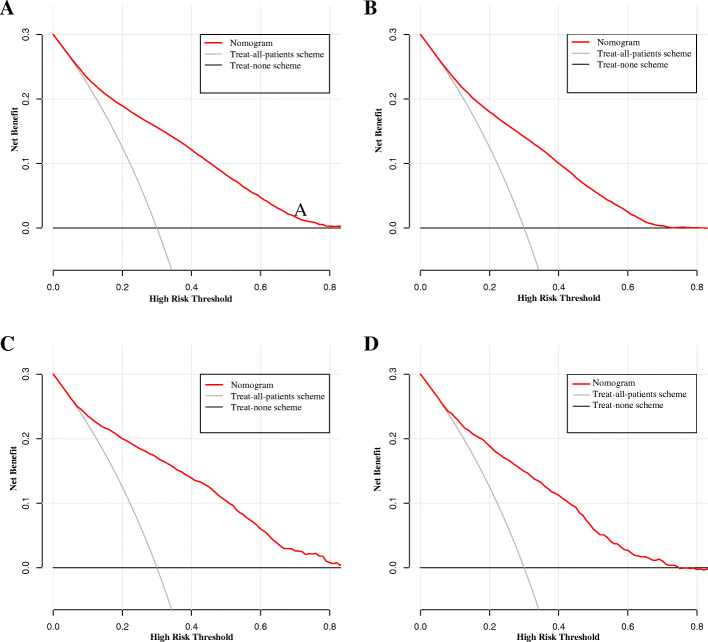
Decision curve analysis (DCA) for the nomogram of (**A**) overall early death in the SEER database diagnosis between 2010 and 2015; (**B**) overall early death in the SEER database diagnosis at 2016; (**C**) cancer-specific early death in the SEER database diagnosis between 2010 and 2015; (**D**) cancer-specific early death in the SEER database diagnosis at 2016

**Fig. 8:**
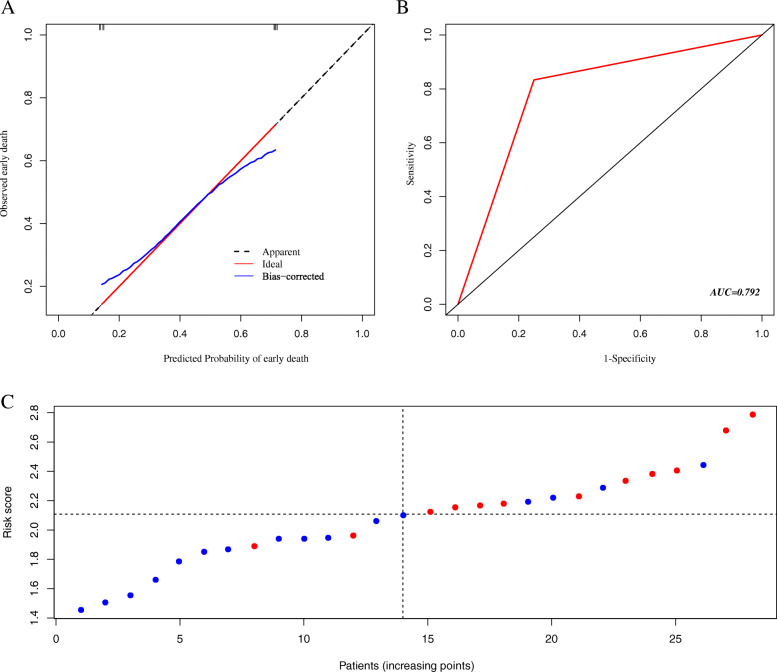
Validation in hospital cohort. (**A**) the calibration plots for the nomogram; (**B**) the ROC curve for the nomogram; (**C**) Total points distribution and early death status of patients in the hospital cohort

The nomogram showed good prediction efficiency on the probability of early death. The ROC curve used to assess the nomogram of overall and cancer-specific early death was shown in Fig. 5. The areas under the curve (AUC) of overall early death was 0.793 (Fig. 5A; 95% CI: 0.788–0.799), while the AUC of cancer-specific early death was 0.794 (Fig. 5B; 95% CI: 0.788–0.799) in the training group. And the AUC in the validation group were 0.803 for overall early death (Fig. 5C; 95% CI: 0.788–0.818) and 0.806 for cancer-specific early death (Fig. 5D; 95% CI: 0.791–0.821), respectively. The calibration plots of the model showed that the predicted early death was consistent with the actual value (Fig. 6). In addition, the DCA analysis indicated a good clinical application value of this model. (Fig. 7). Then, patients were scored by the nomogram in a hospital cohort. We only used the nomogram for cancer-specific early death, since the cause of early death in all patients was lung cancer and related factors. The total score of patients in the hospital cohort ranged from 145 to 279, which we divide them into high-score and low-score groups by setting the cutoff to 210 It showed that the early mortality in high score group was much higher than that in low score group, and the AUC was up to 0.792 (Fig. 8; 95% CI: 0.636–0.947).

### Web-based probability calculator

**Fig. 9:**
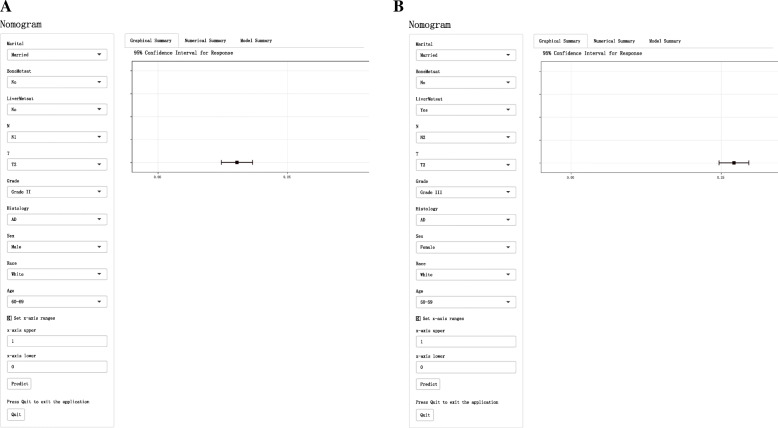
A web-based probability calculator. The graphical summary showed a rough range of overall (**A**) and cancer-specific (**B**) early death probability and its 95% confidence interval

On the basis of the previous nomogram to predict the early death of patients with LCBM, the overall (Fig. 9A) and cancer specific (Fig. 9B) early death probability calculator based on Web was constructed (https://lcbmdynnom.shinyapps.io/lcbmofall/ and https://lcbmdynnom.shinyapps.io/lcbmcss/). The clinical characteristics of patients can be directly input to predict the early death probability of patients.

## Discussion

Brain metastasis is one of the common causes of death in cancer patients, and lung cancer is the main primary tumor of brain metastasis. Nowadays the survival time of patients has been significantly prolonged attributed to the early diagnosis and standardized treatment of lung cancer. However, once brain metastases occur, it will lead to neurological dysfunction, epilepsy and even delirium, which seriously endangers the survival of patients [18]. Thus, brain metastatic from lung cancer has become a major problem in neurosurgery.

The poor prognosis of brain metastatic lung cancer has always been a concern. However, most studies focus on the long-term survival of lung cancer patients with brain metastases, the early death of these patients has not been explored [19–22]. The definition of early death varies from studies and is usually defined as 30 days to 3 months after diagnosis. In this study, early death was defined as 3 months.

This study found that the overall early motality of lung cancer was 27.5%, and the rate increased to 44.4% once brain metastasis occurred, which indicated poor prognosis of lung cancer patients. Although the prognosis of lung cancer patients has improved in recent years, we found that the early mortality of patients with LCBM remained stable from 2010 to 2016, which indicates that we need to pay more attention to early death and related factors to reduce the risk of early death. Further studies found that age, race, gender, Gleason grade, histological type, T stage, N stage, bone metastasis, liver metastasis and marital status were independent risk factors for early death of patients with LCBM. Previous studies has shown that these risk factors have significant impact on the long-term survival of lung cancer patients (except bone metastases to SCLC), while it also shows that they have impact on the early death of patients with LCBM in this study [23].

The study on early death has been applied to advanced cancers in other systems and has shown important clinical significance. Song et al. established a nomogram chart to predict the early mortality of uterine sarcoma, which was significantly better than FIGO stage system [24]. Yang et al. established a model to predict the early mortality of stage IV gastric cancer, and the AUC was as high as 0.847 [25]. These studies demonstrate the feasibility and significance of nomograms in predicting early cancer motality. In our study, we established nomograms of overall and cancer-specific early death probability, according to the risk factors obtained from logistic regression analysis. The nomograms showed good predictive ability and clinical applicability. Internal validation of the nomogram showed good agreement between the predicted early deaths and the actual ones. DCA curves showed that our nomograms have good clinical value and practicability in predicting survival rate. It could provide a portable early death screening and clinical decision-making tool for clinicians, so as to customize the targeted therapy after diagnosis with LCBM.

This study also has several limitations. First of all, there is no information on molecular pathological indicators in SEER data set, and there are no positive prognostic variables. These variables may be an effective supplement to the existing system, which will be the main part of our future research. In addition, some indicators related to patients’ basic information, such as comorbidity rate, were not included in the study. In addition, although external validation is carried out, the amount of data is small, and the model still needs external validation of larger samples to estimate the accuracy.

## Conclusion

In conclusion, we established a comprehensive nomogram to predict early death in lung cancer patients with synchronous brain metastases. Nomograms may help oncologists develop better treatment strategies, such as clinical trials and hospice care.

## Supplementary Information

**Additional file 1.**

## Data Availability

The raw data of this study are derived from the SEER database, which is a publicly available database. The SEER detailed data of SEER database included in the study are available to all at https://seer.cancer.gov/. The hospital cohort database was available in the supplementary materials. More specific data used during the present study are available from the corresponding author upon reasonable request.
